# The effect of α-lactalbumin consumption on sleep quality and quantity in female rugby union athletes: a field-based study

**DOI:** 10.5114/biolsport.2023.116002

**Published:** 2022-06-01

**Authors:** Madeleine Gratwicke, Kathleen H Miles, Brad Clark, Kate L Pumpa

**Affiliations:** 1 Discipline of Sport and Exercise Science, University of Canberra, Canberra, Australia; 2 Research Institute for Sport and Exercise, University of Canberra, Canberra, Australia

**Keywords:** Team-sport, Nutritional Interventions, Tryptophan, Recovery, Protein, Wrist Actigraphy

## Abstract

This double-blind randomised placebo-controlled trial aimed to investigate the effects of α-lactalbumin consumption on sleep quality and quantity in female rugby union athletes during a competition season. Eighteen semi-professional female rugby union players (age 23.8 ± 5.2 y; mean ± SD) wore wrist actigraphy watches for four seven-day blocks corresponding to the pre-season, a home game, a bye week (i.e. no competition game scheduled) and an away game. Participants consumed either an α-lactalbumin (α-LAC), or placebo drink (PLA) every night two hours before bed for the duration of the season. Generalised linear mixed models were used to investigate the effects of the nutritional intervention on sleep variables (total sleep time, sleep efficiency (SE), sleep onset latency (SOL) and wake after sleep onset) over the duration of the season. There was a significant condition by period interaction effect on SOL (p = 0.01). While similar at baseline (23.3 ± 16.3 and 23.2 ± 18.9 min α-LAC and placebo respectively) and for the home game (22.4 ± 17.6 and 19.3 ± 14.9 min α-LAC and placebo respectively), SOL was reduced in the α-LAC group for the bye (11.6 ± 13.4 min) and away game (17.0 ± 11.5 min; p = 0.045). In comparison, SOL remained unchanged in the PLA group (bye 21.2 ± 17.3 and away 22.5 ± 18.5 min). Pre-sleep α-lactalbumin consumption improved SOL in a semi-professional female team-sport cohort. Thus, α-lactalbumin could be utilised by athletes to support sleep during a competitive season.

## INTRODUCTION

Sleep is widely recognised as being critical to athlete recovery and optimal performance. However, athletes appear to experience more sleep disturbances than the general population [1, 2] relating to training, travel, and competition commitments [3, 4]. Despite clear evidence supporting the importance of restorative sleep [5], the efficacy of interventions to negate disturbances and improve sleep and related recovery outcomes in athletes is somewhat equivocal. While research suggests both sleep hygiene education and sleep extension interventions are effective short-term, the sustainability of their effects is questionable without ongoing follow up and further education sessions [6]. Furthermore, a recent survey study by Miles et al. [7] indicated female athletes may be resistant or less likely to implement sleep hygiene interventions. As such, additional, easily implemented techniques to improve sleep in the female athlete population are required.

One candidate for an easily implemented intervention is nutrient supplementation, with emerging evidence suggesting that nutritional supplements may be able to improve sleep [8–11]. Candidate supplements from studies in general population cohorts include tart cherry juice and kiwifruit, both of which show modest beneficial effects on sleep parameters due to their natural level of both melatonin and seratonin [12–14]. Importantly, many athletes may already have a supplement regimen with research suggesting 40–70% of athletes use protein supplements [15, 16]. As such, altering existing protein supplement protocols may offer a more feasible and easily implemented nutritional intervention to improve sleep. Recent studies suggest pre-sleep protein ingestion can improve physical recovery processes (e.g. muscle repair and immune function) and there is evidence that certain proteins may improve sleep outcomes [17, 18].

A protein which has recently been investigated as a nutritional pre-sleep intervention is α-lactalbumin, which is the dominant protein in human milk and constitutes approximately 17% of whey proteins in bovine milk [19, 20]. α-lactalbumin is reported to have the highest natural level of tryptophan [20], which is an essential amino acid and serves as a precursor to the synthesis of the sleep promoting hormones of serotonin and melatonin [21]. Ong et al. [22] reported improvements in sleep quantity and quality for healthy adult participants following 20 g of α-lactalbumin supplementation. Additionally, findings from Miles et al. [23] indicate acute pre-sleep supplementation of α-lactalbumin in a trained female cohort may improve parameters of sleep following simulated evening competition. However, while some evidence suggests α-lactalbumin may be a useful intervention to improve sleep, this is yet to be evaluated in free living conditions in a competitive athlete population.

Thus, we aimed to investigate the effects of α-lactalbumin consumption on sleep quality and quantity in semi-professional female rugby union players during a competition season. We hypothesised that the ingestion of the α-lactalbumin supplement would improve markers of sleep quality and quantity.

## MATERIALS AND METHODS

### Participants

A priori power analysis using female sleep data collected from wrist activity monitors and the Harvard sample size calculator determined that 14 participants would be sufficient to detect a significant medium to large change of f^2^ = 0.25 in sleep outcome variable total sleep time (TST) at an alpha = 0.05 and power = 0.80 [24]. Twenty semi-professional female rugby union athletes (23.8 ± 5.2 years) provided written informed consent to participate in this study. The participants were part of a Super Women’s rugby team and were well-trained, and free of any injuries, diagnosed sleep conditions, illnesses or intolerances (i.e. lactose and gluten) that would exclude them from participating. Training structure during the study period was the same for all participants and consisted of three field sessions, two gym sessions and one recovery session in the pool per week. The study was approved by the Human Research Ethics Committee of the University of Canberra (HREC 2297).

### Experimental overview

**FIG. 1:**
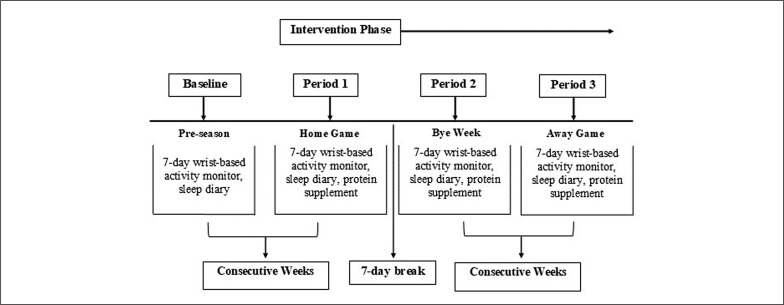
Overview of the study experimental design.

We used a double blinded placebo-controlled trial whereby participants were stratified based on age and playing position (forward or back), then randomly allocated to receive either the α-lactalbumin supplement (α-LAC) or a placebo supplement (PLA, whey protein drink) while their sleep was monitored during a Women’s Super Rugby competition season. The data collection period encompassed four, seven-day blocks corresponding to the pre-season (week prior to competition), a home game (week 1 of competition), a bye week (i.e. no competition game scheduled, week 2 of competition) and an away game (week 3 of competition (Figure 1). The competition then ceased, as did our data collection due to COVID-19.

### Measurement of sleep

Participants wore an activity monitor (Actiwatch 2, Philips Respironics, Pennsylvania, USA) on the wrist of their non-dominant hand which was utilised to assess sleep. Data for each separate week (pre-season, a home game, a bye week and an away game) was collated and averaged for each individual participant for each 7-day period. Validation studies comparing wrist activity monitors with the gold standard polysomnography (PSG) report high correlations for sleep duration (0.84–0.89) and moderate to high correlations for wake time (0.65–0.76) [25]. According to previously described methods from Sargent and colleagues [26], data from the sleep diaries (detailed below) and activity monitors were used to determine when participants were awake and asleep. Time records were scored as awake unless: the sleep diary indicated the participant was lying down attempting to sleep; and monitors activity counts were sufficiently low to indicate that the participant was immobile. Time was scored as sleep when these two conditions were satisfied. This scoring process was conducted using Philips Respironics’ Actiwatch Algorithm with sensitivity set at ‘medium’ to generate measures of total sleep time (TST, sleep duration), sleep efficiency (SE, the percentage of time spent in bed actually spent asleep), sleep onset latency (SOL, time taken to fall asleep) and wake after sleep onset (WASO, time spent awake after falling asleep).

Simultaneously to wearing the activity monitor, participants completed a sleep diary. This sleep diary included before and after sleep sections, where bedtime and wake time are recorded. This data was used in conjunction with the actigraphy data to determine sleep and wake periods.

### Nutritional intervention

The first seven-day block corresponding with the pre-season served as a baseline period and was completed a week prior to the first competition game to assess habitual sleep without the consumption of a protein drink. Following the baseline week (i.e. week of the first competition game), each participant was provided with a seven-day supply of either the α-LAC or PLA in individual containers, with instructions to add 300 mL of water and consume approximately two hours prior to going to bed. At seven-day intervals throughout the trial, the participants were provided with their next supply of the α-LAC or PLA supplement. Any containers not utilised were recorded as an assessment of compliance. This protocol continued for five consecutive weeks from the commencement of the competition season up to the premature end of the season ahead of the grand final due to COVID-19.

Participants randomly assigned to the α-LAC group consumed an α-lactalbumin drink which contained 40 g of α-lactalbumin protein powder (4.8 tryptophan/100 g amino acids) (Davisco Foods International, Eden Prairie, MN, USA) mixed with 10 g of a sugar free drinking chocolate powder (Avalanche, Auckland, New Zealand), 7 g of stevia and 300 mL of water. Those in the PLA consumed a whey protein drink which contained 40 g of chocolate flavoured protein powder (1.05 g tryptophan/100 g) (Body Science Whey Ultra, Burleigh, Australia) and 300 mL of water. A 40 g dose of α-lactalbumin protein powder was chosen based on the two previous studies utilising α-LAC in athletic populations [27, 28]. Both products were batch tested for banned substances by Human and Supplement Testing Australia (HASTA) and were closely matched for colour, texture and flavour. A comparative nutritional analysis can be seen in Table 1.

**TABLE 1:** Nutritional analysis comparing interventions: α-LAC and PLA.

	α-LAC (50 g)	PLA (40 g)

Ingredients (g)	A-LAC protein (40)	Whey protein (40)
**Powdered chocolate (6)**		
**Stevia (4)**		
Energy (kcal)	153	151

**Amino acids (g)**		
Isoleucine	2.4	1.7
Leucine	4.3	3.2
Phenylalanine	1.6	0.9
Tyrosine	1.8	0.9
Valine	1.7	1.7
Tryptophan	1.9	0.4

### Statistical analysis

Sleep data from the activity monitors were included in the data analysis if the participant had worn the monitor for a minimum of 4 days out of the assessable 7 day period, and was scorable using the diaries and watches when cross referenced. All statistical analysis was conducted in *R* Studio statistical software (V 4.0.2, R Studio Inc, Boston, USA). Generalised linear mixed models were used to determine the effect of the nutritional intervention and period of the season on outcome sleep variables. The nutritional intervention (i.e. α-LAC or PLA) and period (i.e. baseline, home game, bye week or away game), and their interaction, were fitted as fixed effects (with some models fitting participant as a random effect with condition and period as random slopes) to determine whether there was a difference in the effect of dietary intervention over period on the dependent sleep variables (e.g. TST, SE, SOL and WASO). All variables are reported a mean ± SD and the significance level at *p* < 0.05.

## RESULTS

Sixteen participants provided adequate sleep data during the baseline week (8 in each group), 15 for the week preceding the home game (α-LAC = 7, PLA = 8), 12 for the week preceding bye week (α-LAC = 5, PLA 7) and 12 for the week ahead of the away game (α-LAC = 5, PLA = 7). During the first week of the nutritional intervention, two nights worth of protein (2 individual serves) were returned within the individual containers provided to each athlete from participants randomised to the α-LAC group. From that point onwards, all α-LAC or PLA containers were returned empty, implying compliance with the nutritional intervention. Bed and wake times for each condition over the season is shown in Table 2. While bed and wake times were similar across the two conditions, participants in the α-LAC group on average went to bed earlier (23:20) compared to PLA (23:52), and woke earlier (α-LAC, 06:42; PLA, 06:57).

**TABLE 2:** Bed and wake times across the season for both conditions

	A-LAC	PLACEBO
**Bedtime**		
Baseline	23:13 ± 01:13	23:29 ± 01:13
Home game	23:17 ± 01:38	24:17 ± 01:46
Bye week	23:14 ± 01:14	23:27 ± 01:11
Away game	23:34 ± 01:38	23:35 ± 01:22

**Wake time**		
Baselin	06:22 ± 01:07	06:26 ± 01:46
Home game	06:37 ± 01:18	07:04 ± 01:24
Bye week	06:51 ± 01:36	06:48 ± 01:40
Away game	06:59 ± 01:42	06:51 ± 01:35

**FIG. 2:**
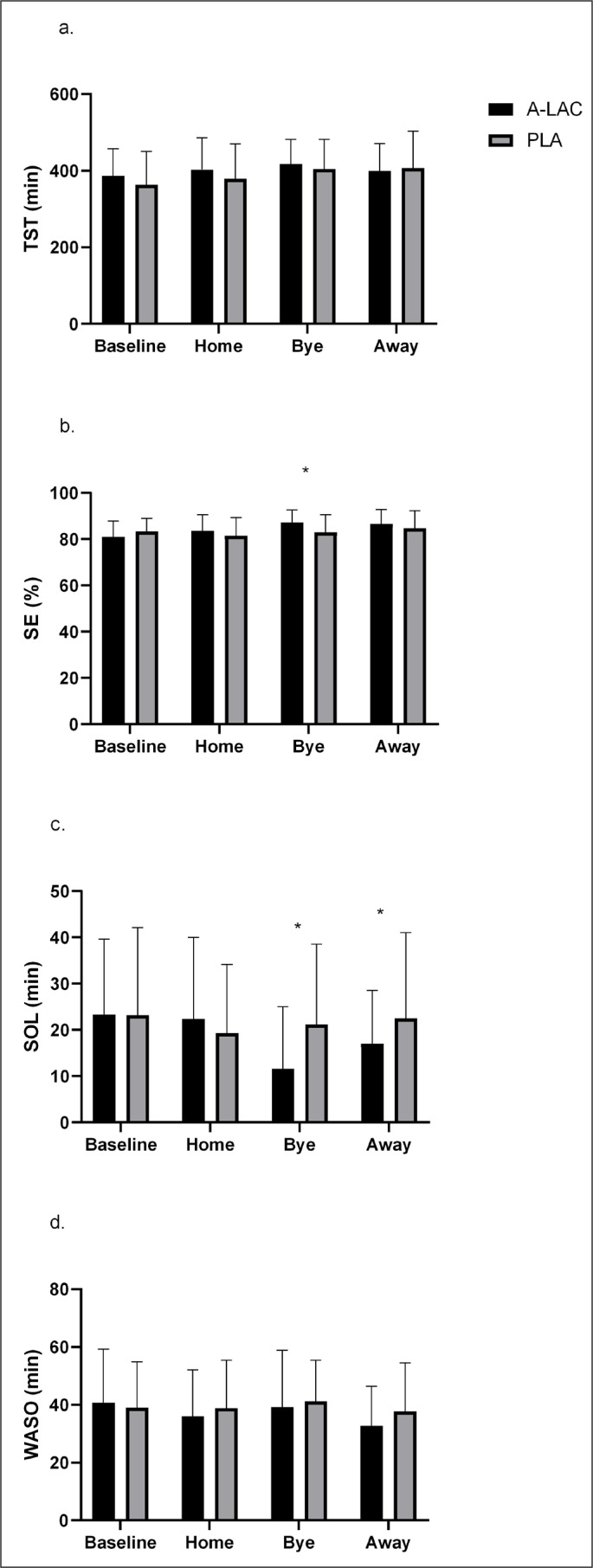
The effects of the nutritional interventions on indices of sleep quantity and quality across the competitive season. *significantly different compared to baseline and placebo. SE = sleep efficiency; TST = total sleep time; SOL = sleep onset latency; WASO = wake after sleep onset.

There was no effect of nutritional supplement on TST or WASO across the intervention period (p > 0.05, Figure 2). However, there was a significant condition by period interaction effect on SOL (p = 0.01). While similar at baseline and for the home game, SOL was significantly shorter in the α-LAC group for the bye week and away game (difference bye week, 9 min; difference away game 5 min, ES = 0.62 and ES = 0.37, p = 0.045) but remained stable in the PLA group. No other significant differences were identified between groups for any other parameter.

## DISCUSSION

The primary purpose of this study was to evaluate the efficacy of α-lactalbumin supplementation to improve sleep quality and quantity in female rugby union players. While we observed no effect of α-lactalbumin supplementation on sleep quantity, in line with our hypothesis α-lactalbumin supplementation led to improvements in SOL with a trend towards improved SE relative to the placebo supplement.

To our knowledge, we are the first to examine the efficacy of α-lactalbumin supplementation as a home-based sleep intervention designed to improve sleep quality and quantity for team-sport athletes. The main finding of our study was that α-lactalbumin supplementation significantly improved SOL and showed a trend towards a significant increase in SE, consistent with reports on the effects of pre-sleep α-lactalbumin ingestion (20 g, an hour prior to bed) in healthy males [22]. Results regarding the efficacy of α-lactalbumin supplementation, however, vary between studies depending on dosage and intervention length. Recently, MacInnis et al. [27] investigated α-lactalbumin supplementation to improve sleep in six elite male cyclists, finding no significant differences between their two conditions (α-lactalbumin and collagen peptides) for any actigraphy recorded sleep variables. However, MacInnis et al. only administered the α-lactalbumin supplement for three days, potentially not long enough to see an effect on sleep outcomes in free living conditions. Indeed, in our study the effects of α-lactalbumin on sleep quality only became evident after several weeks of supplementation, which may indicate there is a cumulative effect of chronic α-LAC consumption. Importantly, given our finding it’s unlikely that α-lactalbumin will negatively affect athlete sleep. Furthermore, given that α-lactalbumin is a source of whey protein which supports muscle remodelling and immune function [18], it appears that α-lactalbumin offers an intervention that supports athlete sleep, and subsequently wellbeing and performance. Future research should investigate the most effective dosage and loading protocol that may positively influence sleep.

When compared to the reported impacts of sleep hygiene strategies on sleep in female athletes, our results suggest α-lactalbumin may have a greater impact on sleep quality. O’Donnell et al. [29] found a sleep hygiene education session resulted in no significant improvements in both SOL and SE. While other studies suggest sleep hygiene may be beneficial in male athlete groups [6, 30], it is yet to be proven whether these protocols can be beneficial in female populations who appear to be more resistant to these interventions than their male counterparts [7]. Thus, α-lactalbumin may be a good addition to other sleep interventions that may not be beneficial alone. α-lactalbumin may also serve as an alternative to pharmaceutical aids such as temazepam, melatonin, zaleplon, and zopiclobe [31, 32], which are known to improve sleep, but are associated with other negative outcomes such as performance decrements, dependency, possible increased cancer risk, accidental overdose, and dangerous drug interactions [33]. Future research should investigate α-lactalbumin supplementation in conjunction with interventions like sleep hygiene/ extension to substantially improve sleep more so than either intervention could alone.

As our study was conducted in free living conditions, there were no restrictions placed on participant bedtime habits or daytime naps, and as such, TST was limited by the time our participants had available in bed around late and early training sessions, and did not include daytime naps. While we saw a significant difference in SOL, the limited time in bed perhaps wasn’t enough to see an increase in TST. In a population where TST may be limited, the ability of a nutritional intervention to enhance SOL and SE would be valuable, particularly female athletes who often train around full-time work and family commitments. A study investigating the sleep behaviours of elite swimmers found TST and SE were substantially poorer on training days compared to a rest day [34]. Similar findings were reported in a mixed cohort of elite and sub-elite Irish athletes, with both elite and sub-elite athletes reporting poor sleep quality, which improved on rest days [35]. Female team-sport athletes also often face undesirable training schedules (i.e. morning or late evening sessions) which can compromise an optimal recovery regime by reducing sleep quantity due to limited time in bed [36]. Thus, the efficacy of α-lactalbumin supplementation to improve sleep quality regardless of time available in bed could be highly beneficial for team-sport athletes.

Although our study did not investigate the mechanisms by which α-lactalbumin supplementation may influence sleep outcomes, earlier work in general populations suggest beneficial effects on sleep may be due to the loading of tryptophan, an essential amino acid that serves as a precursor to the sleep promoting hormones melatonin and serotonin [21]. Tryptophan is reported to have beneficial effects on sleep indices, increasing the total sleep time and reducing waking time encountered by individuals [19]. Pre-sleep administration of tryptophan increases the plasma tryptophan to large neural amino acid ratio (TRP:LNAA) [21, 37, 38], leading to an increase in brain tryptophan availability [38]. This is thought to lead to a downstream increase in synthesis of melatonin and serotonin, both which display sedative effects [39, 40]. Although not directly investigating sleep parameters, the research by Markus et al. [37] indicates sleep quality may have improved with a significant reduction in sleepiness, and improved attention processes the morning following the intervention [37]. Markus et al. [38] also reported decreases in depressive feelings following α-lactalbumin consumption in their participants. Research suggests there are strong links between poor sleep and feelings of anxiety and depression, with women experiencing more insomnia complaints than males [41]. As such, an intervention like α-lactalbumin that can increase sleep quality and decrease depressive feelings may be beneficial during high-stress team-sport seasons, particularly for female athletes.

We acknowledge there were several limitations associated with our study, many due to the applied nature of the project that should be taken into consideration when assessing the results. These limitations include: inability to accurately capture training loads due to non-compliance with recording gym sessions and wearing global positioning systems, inability to control for hormonal related factors and menstrual cycle, inability to control nutritional intake, specifically prior to bed which may influence the quality and quantity of the individuals sleep [11], external situational factors, and the number of athletes who participated for the entire duration of the project. The power analysis conducted for this study indicated 14 participants were required to determine significant changes in TST. Although this number was met, we recognise the small sample size of our study may have hindered the statistical power and minimised optimal stratification of participants by playing position. Due to the length of our study and the associated uncontrollable external factors associated with being a semi-professional female athlete, we recommend future studies assessing the efficacy of α-lactalbumin utilise a more intensive or controlled environment like a training camp (whilst acknowledging these settings may see increases in training load and potential implications associated with changes to sleep environments).

## CONCLUSIONS

In conclusion, this study provides initial field-based evidence on the benefits of α-lactalbumin consumption across a competitive season. This study highlights the efficacy of pre-sleep α-lactalbumin consumption as a nutritional intervention to improve SOL in a semi-professional female team-sport cohort. Thus, α-lactalbumin could be utilised by athletes to support sleep during a competitive season and be taken easily in the home-environment regardless of training or lifestyle requirements.

## References

[1] Fullagar H, Skorski S, Duffield R, Hammes D, Coutts A, Meyer T. Sleep and athletic performance: the effects of sleep loss on exercise performance, and physiological and cognitive responses to exercise. Sports Med. 2015; 45(2):161–186. 25315456 10.1007/s40279-014-0260-0

[2] Knufinke M, Nieuwenhuys A, Geurts SA, Møst EI, Maase K, Moen MH, Coenen AM, Kompier MA. Train hard, sleep well? Perceived training load, sleep quantity and sleep stage distribution in elite level athletes. J Sci Med Sport. 2018; 21(4):427–432. 28754605 10.1016/j.jsams.2017.07.003

[3] Walsh NP, Halson SL, Sargent C, Roach GD, Nédélec M, Gupta L, Leeder J, Fullagar HH, Coutts AJ, Edwards BJ. Sleep and the athlete: narrative review and 2021 expert consensus recommendations. Br J Sports Med. 2021; 55(7):356–368. 10.1136/bjsports-2020-102025 33144349

[4] Gupta L, Morgan K, Gilchrist S. Does elite sport degrade sleep quality? A systematic review. Sports Med. 2017; 47(7):1317–1333. 27900583 10.1007/s40279-016-0650-6 PMC5488138

[5] Tuomilehto H, Vuorinen V-P, Penttilä E, Kivimäki M, Vuorenmaa M, Venojärvi M, Airaksinen O, Pihlajamäki J. Sleep of professional athletes: underexploited potential to improve health and performance. J Sports Sci. 2017; 35(7):704–710. 27173843 10.1080/02640414.2016.1184300

[6] Caia J, Scott TJ, Halson SL, Kelly VG. The influence of sleep hygiene education on sleep in professional rugby league athletes. Sleep Health. 2018; 4(4):364–368. 30031530 10.1016/j.sleh.2018.05.002

[7] Miles KH, Clark B, Fowler PM, Miller J, Pumpa KL. Sleep practices implemented by team sport coaches and sports science support staff: A potential avenue to improve athlete sleep? J Sci Med Sport. 2019; 22(7):748–752. 30685228 10.1016/j.jsams.2019.01.008

[8] Leonarda G, Fedele E, Vitale E, Lucini D, Mirela V, Mirela IA. Healthy athlete’s nutrition. Medicina Sportiva: Journal of Romanian Sports Medicine Society. 2018; 14(1):2967–2985.

[9] Mahurkar A. Importance of balance diet & nutrition for athletes performance. Indian J Appl Res. 2019; 9(11).

[10] Doherty R, Madigan S, Warrington G, Ellis J. Sleep and nutrition interactions: implications for athletes. Nutrients. 2019; 11(4):822. 30979048 10.3390/nu11040822 PMC6520871

[11] Gratwicke M, Miles KH, Pyne DB, Pumpa KL, Clark B. Nutritional Interventions to Improve Sleep in Team-Sport Athletes: A Narrative Review. Nutrients. 2021; 13(5):1586. 34068512 10.3390/nu13051586 PMC8150598

[12] Lin H-H, Tsai P-S, Fang S-C, Liu J-F. Effect of kiwifruit consumption on sleep quality in adults with sleep problems. Asia Pac J Clin Nutr. 2011; 20(2):169. 21669584

[13] Howatson G, McHugh MP, Hill JA, Brouner J, Jewell AP, Van Someren KA, Shave RE, Howatson SA. Influence of tart cherry juice on indices of recovery following marathon running. Scand J Med Sci Sports. 2010; 20(6):843–852. 19883392 10.1111/j.1600-0838.2009.01005.x

[14] Pigeon WR, Carr M, Gorman C, Perlis ML. Effects of a tart cherry juice beverage on the sleep of older adults with insomnia: a pilot study. J Med Food. 2010; 13(3):579–583. 20438325 10.1089/jmf.2009.0096 PMC3133468

[15] Aguilar-Navarro M, Baltazar-Martins G, Brito de Souza D, Muñoz-Guerra J, del Mar Plata M, Del Coso J. Gender differences in prevalence and patterns of dietary supplement use in elite athletes. Res Q Exerc Sport. 2020:1–10. 10.1080/02701367.2020.1764469 32809924

[16] Garthe I, and Ramsbottom R. Elite athletes, a rationale for the use of dietary supplements: a practical approach. PharmaNutrition. 2020:100234.

[17] Abbott W, Brett A, Cockburn E, Clifford T. Presleep casein protein ingestion: acceleration of functional recovery in professional soccer players. Int J Sports Physiol Perform. 2018; 14(3):385–391. 10.1123/ijspp.2018-0385 30204517

[18] West DW, Abou Sawan S, Mazzulla M, Williamson E, Moore DR. Whey protein supplementation enhances whole body protein metabolism and performance recovery after resistance exercise: A double-blind crossover study. Nutrients. 2017; 9(7):735. 28696380 10.3390/nu9070735 PMC5537849

[19] Layman DK, Lönnerdal B, Fernstrom JD. Applications for α-lactalbumin in human nutrition. Nutr Rev. 2018; 76(6):444–460. 29617841 10.1093/nutrit/nuy004 PMC5934683

[20] Rozé J-C, Barbarot, S, Butel, M-J, Kapel, N, Waligora-Dupriet, A-J, De Montgolfier, I, Leblanc, M, Godon, N, Soulaines, P, Darmaun, D. An α-lactalbumin-enriched and symbiotic-supplemented v. a standard infant formula: a multicentre, double-blind, randomised trial. Br J Nutr. 2012; 107(11):1616–1622. 22079177 10.1017/S000711451100479X

[21] Heine W, Radke M, Wutzke K-D. The significance of tryptophan in human nutrition. Amino Acids. 1995; 9(3):91–205. 24178836 10.1007/BF00805951

[22] Ong JN, Hackett DA, Chow C-M. Sleep quality and duration following evening intake of alpha-lactalbumin: a pilot study. Biol Rhythm Res. 2017; 48(4):507–517.

[23] Miles K, Clark, B, Fowler, PM, Gratwicke, MJ, Martin, K, Welvaert, M, Miller, J, Pumpa, KL. ɑ-lactalbumin Improves Sleep and Recovery Post Simulated Evening Competition in Female Athletes. Med Sci Sports Exerc. 2021. 10.1249/MSS.0000000000002743 34649262

[24] Schoenfeld D. Statistical considerations for clinical trials and scientific experiments. 2015 [cited 2019 December]; Available from: http://hedwig.mgh.harvard.edu/sample_size/size.html

[25] Weiss AR, Johnson NL, Berger NA, Redline S. Validity of activity-based devices to estimate sleep. J Clin Sleep Med. 2010; 6(4):336–342. 20726281 PMC2919663

[26] Sargent C, Lastella M, Halson SL, Roach GD. The validity of activity monitors for measuring sleep in elite athletes. J Sci Med Sport. 2016; 19(10):848–853. 26794719 10.1016/j.jsams.2015.12.007

[27] MacInnis MJ, Dziedzic CE, Wood E, Oikawa SY, Phillips SM. Presleep α-Lactalbumin Consumption Does Not Improve Sleep Quality or Time-Trial Performance in Cyclists. Int J Sport Nutr Exerc Metab. 2020; 30(3):197–202. 32698123 10.1123/ijsnem.2020-0009

[28] Oikawa SY, Macinnis MJ, Tripp TR, McGlory C, Baker SK, Phillips SM. Lactalbumin, Not Collagen, Augments Muscle Protein Synthesis with Aerobic Exercise. Med Sci Sports Exerc. 2019. 10.1249/MSS.0000000000002253 31895298

[29] O’Donnell S, and Driller MW. Sleep-hygiene education improves sleep indices in elite female athletes. Int J Exerc Sci. 2017; 10(4):522. 28674597 10.70252/DNOL2901 PMC5466408

[30] Driller MW, Lastella M, Sharp AP. Individualized sleep education improves subjective and objective sleep indices in elite cricket athletes: A pilot study. J Sports Sci. 2019; 37(17):2021–2025. 10.1080/02640414.2019.1616900 31076021

[31] Paul MA, Gray G, MacLellan M, Pigeau RA. Sleep-inducing pharmaceuticals: a comparison of melatonin, zaleplon, zopiclone, and temazepam. Aviat Space Environ Med. 2004; 75(6):512–519. 15198277

[32] Reilly T, Atkinson G, Budgett R. Effect of low-dose temazepam on physiological variables and performance tests following a westerly flight across five time zones. Int J Sports Med. 2001; 22(03):166–174. 11354518 10.1055/s-2001-16379

[33] Taylor L, Chrismas BC, Dascombe B, Chamari K, Fowler PM. Sleep medication and athletic performance—the evidence for practitioners and future research directions. Front Physiol. 2016; 7:83. 27014084 10.3389/fphys.2016.00083 PMC4779957

[34] Sargent C, Halson S, Roach GD. Sleep or swim? Early-morning training severely restricts the amount of sleep obtained by elite swimmers. Eur J Sport Sci. 2014; 14(sup1):S310–S315. 24444223 10.1080/17461391.2012.696711

[35] Doherty R, Madigan SM, Nevill A, Warrington G, Ellis JG. The sleep and recovery practices of athletes. Nutrients. 2021; 13(4):1330. 33920560 10.3390/nu13041330 PMC8072992

[36] Sargent C, Lastella M, Halson SL, Roach GD. The impact of training schedules on the sleep and fatigue of elite athletes. Chronobiol Int. 2014; 31(10):1160–1168. 25222347 10.3109/07420528.2014.957306

[37] Markus CR, Jonkman LM, Lammers JH, Deutz NE, Messer MH, Rigtering N. Evening intake of α-lactalbumin increases plasma tryptophan availability and improves morning alertness and brain measures of attention. Am J Clin Nutr. 2005; 81(5):1026–1033. 15883425 10.1093/ajcn/81.5.1026

[38] Markus C, Olivier, B, Panhuysen, GEM, Van der Gugten, J, Alles, MS, Tuiten, A, Westenberg, HGM, Fekkes, D, Koppeschaar, HF, de Haan, EEHF. The bovine protein α-lactalbumin increases the plasma ratio of tryptophan to the other large neutral amino acids, and in vulnerable subjects raises brain serotonin activity, reduces cortisol concentration, and improves mood under stress. Am J Clin Nutr. 2000; 71(6):1536–1544. 10837296 10.1093/ajcn/71.6.1536

[39] Kokturk O, and Kanbay A, Tryptophan metabolism and sleep, in Tryptophan Metabolism: Implications for Biological Processes, Health and Disease, Engin A, Engin, Ayse Basak, Editor. 2015, Springer: Switzerland. p. 239–252.

[40] Silber B, and Schmitt J. Effects of tryptophan loading on human cognition, mood, and sleep. Neurosci Biobehav Rev. 2010; 34(3):387–407. 19715722 10.1016/j.neubiorev.2009.08.005

[41] Spoormaker VI, and van den Bout J. Depression and anxiety complaints; relations with sleep disturbances. Eur Psychiatry. 2005; 20(3):243–245. 15935423 10.1016/j.eurpsy.2004.11.006

